# Strengthening and expanding health inequality monitoring for the advancement of health equity: a review of WHO resources and contributions

**DOI:** 10.1186/s12939-022-01811-4

**Published:** 2023-03-17

**Authors:** Ahmad Reza Hosseinpoor, Nicole Bergen, Katherine Kirkby, Anne Schlotheuber

**Affiliations:** Department of Data and Analytics, World Health Organization, 20 Avenue Appia, CH-1211 Geneva 27, Switzerland

**Keywords:** Capacity building, Disaggregated data, Health equity, Health inequality, Monitoring, World Health Organization

## Abstract

As part of its commitment to advance health equity, the World Health Organization (WHO) has a developed area of work to promote and strengthen health inequality monitoring. This includes an emphasis on the collection, analysis and use of disaggregated health data, which are central to evidence-informed decision making. The aim of this paper is to review WHO's work on health inequality monitoring, namely the *2022–27 Inequality monitoring and analysis strategy* and corresponding activities, resources and tools. The strategy has three goals pertaining to: strengthening capacity for health inequality monitoring; generating and disseminating the latest evidence on health inequality and supporting data disaggregation; and developing and refining health inequality monitoring methods, resources and best practices. In alignment with these goals, WHO has published reference materials focused on conceptual approaches to health inequality monitoring, which are applied in the global State of Inequality report series. The Health Inequality Monitoring eLearning channel on OpenWHO and capacity building workshops and webinars facilitate the uptake and application of inequality monitoring practices across diverse settings and stakeholders. A key tool available to support the analysis and reporting aspects of health inequality monitoring is the Health Equity Assessment Toolkit (HEAT) application, which allows users to explore data interactively. The Health Inequality Data Repository, a collection of the largest publicly available database of disaggregated data from around the globe, further enables inequality monitoring and analyses. This collection of resources is an important contribution to promote health inequality monitoring across diverse settings. The uptake of evidence from health inequality monitoring remains crucial to the advancement of equity as part of global health and development initiatives.

**Supplementary Information:**

The online version contains supplementary material available at 10.1186/s12939-022-01811-4.

## Introduction

*“The gradual realization of the potentialities of the scientific and social techniques available […] linked with the growing sense of the community’s responsibilities toward the underprivileged – have led to the broad social and humanitarian viewpoint embodied in the constitution of the World Health Organization”* [1]

-Brock Chisholm, first Director General of the World Health Organization [1]

The amelioration of social inequalities in health has been acknowledged and embedded in the work of the World Health Organization (WHO) since its establishment and remains a priority of the Organization to this day. The adoption of the United Nations 2030 Agenda for Sustainable Development in 2015 is a recent and ongoing commitment to addressing inequalities by WHO and global development partners. Central to the Agenda is a pledge to leave no one behind in pursuit of 17 integrated health and development goals [2]. This is reflected in goal 10, which is dedicated to the reduction of inequalities within and among countries.

Aligned with the health-related indicators set out in the 2030 Agenda, the WHO’s Triple Billion targets aim to improve the health of billions of people over the 2019–2025 period by ensuring that: one billion more people are benefiting from universal health coverage; one billion more people are better protected from health emergencies; and one billion more people are enjoying better health and well-being [3]. The Triple Billion targets are the basis for the measurement and policy strategy of the Organization’s Thirteenth General Programme of Work (GPW 13), which is focused on delivering measurable impact in countries, backed by the highest standards of health data [4]. GPW 13 commits to promoting the "strategic disaggregation of data through collection, analysis and reporting to better inform programmes based on the following: sex, income, disability, ethnicity and age group categories in surveys, routine data and other data sources" [4]. Only half of countries included disaggregation in national health statistics reports according to the 2020 WHO SCORE Global Report, which assessed the status and capacity of health information systems across 133 countries [5].

**Fig. 1:**
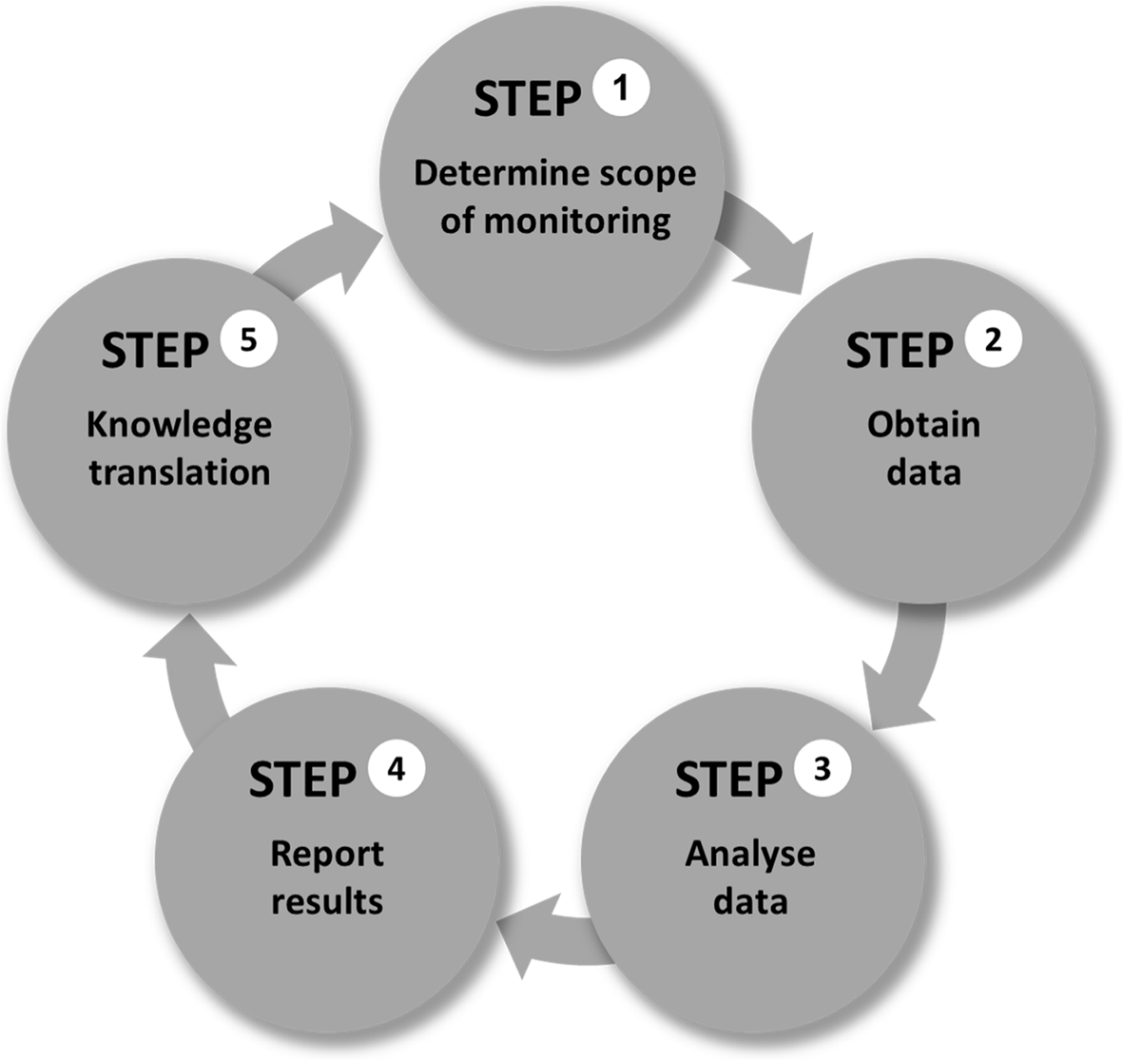
Health inequality monitoring represented as a five-step cycle

The collection, analysis and use of disaggregated health data are at the core of health inequality monitoring. Health inequalities refer to measurable differences in health across population subgroups, defined by social, economic, demographic or geographic characteristics [6]. Monitoring health inequalities is the process of routinely quantifying and assessing health inequalities in a defined population. It can be conceptualized as a five-step cycle, consisting of: (a) determining the scope of monitoring; (b) obtaining disaggregated data; (c) analyzing data; (d) reporting results; and (e) engaging in knowledge translation activities [7] (Fig. 1). The results derived from monitoring provide evidence for evaluating and progressing health equity – the absence of unfair, avoidable or remediable differences in health among population subgroups [8, 9] – and can be used to determine where equity-oriented changes are needed in policies, programmes and practices. This process has applications for researching health inequalities and for the routine monitoring of inequalities as part of health information systems.

WHO supports the establishment and strengthening of sustainable health inequality monitoring systems across diverse settings, health topics, and populations [10]. In this paper, we provide an updated review of WHO efforts in the area of health inequality monitoring, including a recent strategy and current activities, resources and tools. Our objective is to demonstrate how this multifaceted strategy and associated resources can accelerate health inequality monitoring practices among Member States and raise the profile of global evidence on health inequalities.

## Inequality Monitoring and Analysis Strategy

The *2022–27 Inequality monitoring and analysis strategy* was developed by the Health Inequality Monitoring team in consultation with the relevant stakeholders within WHO, with the overarching vision of contributing towards the achievement of health equity by monitoring health inequality, upholding the underlying values of technical excellence and expertise, credibility, diversity and inclusion, collaboration and innovation [11]. Accordingly, the mission is to support Member States with health inequality monitoring and to provide global evidence on health inequality. The strategy has three strategic goals and related objectives, centered around: strengthening capacity for health inequality monitoring; generating and disseminating high quality evidence on health inequality; and developing and refining health inequality monitoring methods, tools, resources and best practices (Table 1).

**Table 1:** Inequality Monitoring and Analysis Strategy goals and objectives

Goal	Strategic objectives
1. Strengthen capacity for health inequality monitoring at country, regional and global levels	1.1: Strengthen capacity for health inequality monitoring among Member States 1.2: Strengthen capacity for health inequality monitoring within the three levels of WHO and other global partners 1.3: Establish and operationalise a global network of international experts for health inequality monitoring
2. Generate and disseminate the latest evidence on health inequality and support the incorporation of data disaggregation within WHO and global partners	2.1: Develop and update the Health Inequality Data Repository and associated dashboards 2.2: Publish and disseminate evidence related to the state of health inequality through reports, journal articles and other publications 2.3: Provide technical support to WHO Headquarters programmes, WHO regions and global partners to incorporate data disaggregation into their databases, monitoring reports and performance measurement
3. Develop and refine health inequality monitoring methods, tools, resources, and best practices	3.1: Develop and advance methods, approaches, tools and resources for health inequality monitoring 3.2: Refine the Health Equity Assessment Toolkit (HEAT and HEAT Plus) and develop related inequality monitoring toolkits

The strategy is grounded in a detailed implementation roadmap, which describes key activities associated with each strategic objective. These activities are tracked on a recurring basis according to indicators laid out in an associated monitoring and evaluation framework. As part of goal one, activities include conducting training workshops with a variety of stakeholder groups, providing technical support for data disaggregation, and establishing a global network of health inequality monitoring experts. Activities related to goal two include the development of the expanded Health Inequality Data Repository (Kirkby K, Bergen N, Baptista A, Schlotheuber A, Hosseinpoor AR: Data resource profile: WHO Health Inequality Data Repository, under review), and the publication of health inequality reports and articles, as well as presentations and webinars, to disseminate evidence on health inequalities. A major activity linked to the third goal is the development of the Health Equity Assessment Toolkit (HEAT) application [12], along with expanding the existing collection of practical resources for health inequality monitoring (including step-by-step manuals, workbooks, eLearning courses and statistical codes).

## WHO activities, resources and tools for health inequality monitoring

### Handbook, step-by-step manuals and workbooks

https://www.who.int/data/inequality-monitor/tools-resources

#### Aim

WHO has developed several resources aimed at providing conceptual guidance for health inequality monitoring. These resources support the widespread implementation of inequality monitoring across different settings and topics. The *Handbook for health inequality monitoring: with a special focus on low- and middle-income countries* and step-by-step manuals and accompanying workbooks provide guidance on the complete cycle of inequality monitoring and its application. These are designed to be highly accessible references to a wide diversity of users.

#### Description

The *Handbook for health inequality monitoring: with a special focus on low- and middle-income countries* introduces key concepts related to health inequality monitoring, illustrated by examples from low- and middle-income countries [6]. The handbook describes the importance of establishing and strengthening inequality monitoring within low- and middle-income countries, with an emphasis on data sources, measures of health inequality and reporting practices.

Step-by-step manuals are organized around the five-step cycle of health inequality monitoring, applied to the context of national monitoring [7], and the topics of immunization [13] and sexual, reproductive, maternal, newborn, child and adolescent health [14]. The manuals break each step of the cycle into sub-steps or components, with key questions, checklists, examples, references and best practices. Accompanying workbooks follow the same general organization, with exercises, guiding questions, and decision-making prompts. Three versions of the workbook are available: a general workbook for health inequality monitoring [15] and companion workbooks to guide inequality monitoring in sexual, reproductive, maternal, newborn, child and adolescent health [16] and HIV, tuberculosis and malaria*.*

#### Application and future plans

These resources may be of interest to anyone seeking a better understanding or reviewing the practice of inequality monitoring. Researchers can use these resources as reference materials to design and conduct studies that integrate health inequality analyses. The resources are designed to be complementary with all other components of the Health Inequality Monitor website, such that they are cross-referenced and can be used together. Language translations of several of these resources are forthcoming.

#### Access

The handbook, step-by-step manuals and workbooks are available from the Tools and resources webpage of the Health Inequality Monitor website [17].

### Health inequality report series

https://www.who.int/data/inequality-monitor/publications

#### Aim

WHO health inequality reports serve as examples of high-quality, detailed technical reports that address health inequality in a given topic at global or regional levels. The reports showcase best practices in data analysis, interpretation and reporting, applying concepts presented in the handbook and collection of step-by-step manuals (as detailed above). They use disaggregated data that are publicly available. In several cases, these reports provide the first systematic global assessment of inequalities pertaining to the topic area, and therefore they constitute a substantive evidence base for the development of policy, programmes and practices, as well as extended research studies.

#### Description

To date, the report series includes four State of Inequality reports [18–21] and one Explorations of Inequality report [22]. State of Inequality reports address two key questions: what is the latest status of inequality? And how have inequalities changed over time? The latest status of inequality is based on the latest available data within a defined time period. Change in inequality over time is assessed based on a comparison of the latest status of inequality with data from a previous point in time. Three of the State of Inequality reports pertain to health topics (reproductive, maternal, newborn and child health; childhood immunization; and HIV, tuberculosis and malaria). The *Explorations of inequality: childhood immunization* report was prepared as an analytical extension of the *State of inequality: childhood immunization* report, focusing on 10 high priority countries. It is an in-depth analysis characterizing the magnitude of inequality in childhood immunization across multiple dimensions of inequality and employing multiple regression analysis to examine the associations between immunization coverage and selected characteristics and to understand compounded vulnerability and advantage.

#### Application and future plans

The State of Inequality and Explorations of Inequality reports are primarily developed for audiences who work with health information systems and have basic skills in interpreting health-related data. This encompasses technical staff (for example, in ministries of health), public health professionals, researchers, and others. The reports are intended to be used by those interested in gaining more exposure to health inequality monitoring practices, and accordingly, can be developed and/or used in conjunction with other health inequality monitoring capacity building resources (including eLearning courses and workshops, as detailed below). Researchers are called upon to build on the findings presented in the reports through follow up quantitative and qualitative studies. Future reports will address inequalities in understudied health topics and dimensions of inequality.

#### Access

Reports and all accompanying materials can be accessed through the Publications webpage of the Health Inequality Monitor website [23].

### eLearning

https://www.who.int/data/inequality-monitor/training

#### Aim

The Health Inequality Monitoring eLearning channel aims to help learners strengthen their conceptual knowledge and practical skills to enhance the practice of health inequality monitoring. The channel is the most comprehensive collection of free, online training materials for strengthening capacity in health inequality monitoring [24]. The channel hosts self-directed courses that help learners build a conceptual understanding of the inequality monitoring process, apply these concepts within different health topics, and develop practical skills to carry out monitoring. When the channel was launched in 2022, it addressed an unmet need for widely available and accessible training that could be used by diverse stakeholders across different settings.

#### Description

The channel consists of three course series. Health inequality monitoring foundations courses introduce the cycle of health inequality monitoring and describe key concepts and best practices. Five foundations courses are available: an overview of health inequality monitoring, and courses on data sources, health data disaggregation, summary measures of health inequality and reporting. A second course series looks at the application of health inequality monitoring within specific health topics, featuring: immunization; HIV, tuberculosis and malaria; and sexual, reproductive, maternal, newborn, child and adolescent health. The third ‘skill building’ course series provides practical guidance for analysis methods and the use of selected software programmes (for example, Excel, R, Stata, as well as HEAT and HEAT Plus). Each course is presented through 4–6 sequential modules and can be completed in approximately 90–120 min.

The Health Inequality Monitoring eLearning channel is hosted by the OpenWHO platform. OpenWHO is a global social learning network that offers free, interactive eLearning courses on a variety of health topics, supporting multiple languages. Key features of the platform help to attract learners and promote their success in health inequality monitoring courses: courses are free of charge; course materials are available in multiple formats and can be downloaded and used offline; learners can navigate selectively through the courses to access materials that meet their immediate needs; discussion forums provide spaces for learner interactions and networking; and verified certificates of achievement are issued to learners who score at least 80% on the graded assessment at the end of the course.

#### Application and future plans

Health Inequality Monitoring eLearning courses were developed for a wide swath of learners from national settings (such as ministries of health or other health institutes, statistical offices, universities and non-governmental organizations), as well as those working at multilateral or inter-governmental organizations. The target audience for the courses includes monitoring and evaluation officers, data analysts, academics and researchers, public health professionals, medical and public health students, and others with a general interest in health data, inequality monitoring and data analysis. While the courses are suitable for anyone wanting to learn more about the topic of health inequality monitoring, certain courses assume a basic understanding of the topic area (in the case of the topic-specific courses) or software programme (in the case of certain skill building courses). The courses are currently available in English (with written transcripts of all audio and audio-visual components), though language translations and integrated subtitles are planned. Periodic course updates and additions are planned to expand the repertoire of learning options. The development of new courses will be aligned, in part, with emerging areas of work.

#### Access

The Health Inequality Monitoring eLearning courses, plus related publications and communications can be accessed through the Training webpage of the Health Inequality Monitor website [25]. The courses can also be accessed through the Health Inequality Monitoring eLearning channel on OpenWHO [26].

### Capacity building workshops

#### Aim

Capacity building workshops aim to establish and strengthen systematic and sustainable approaches to health inequality monitoring at a national level and provide opportunities for professional networking among participants. Workshops focus on theoretical and practical foundations of monitoring, helping participants gain skills in analysis, interpretation and application of health inequality data. Working with existing datasets from the workshop setting(s), participants are exposed to the complexities of health inequality monitoring and encouraged to develop strategies for the practical application of concepts.

#### Description

Workshops are held in person or online, typically spanning 2–4 days, with 20–30 participants. (On occasion, online workshops have been delivered through a condensed format.) The content and format of each workshop is tailored to the needs and priorities of the participating countries. Some workshops have a country or thematic focus, while others have been conducted with participants from a common region or a group of countries. Generally, the workshops consist of presentations, demonstrations, hands-on group activities and plenary discussions, with a heavy focus on the analysis and interpretation of disaggregated data and basic summary measures of inequality using HEAT. Participating countries are encouraged to apply these techniques through the preparation of post-workshop reports about the state of inequality. They are also encouraged to promote the practice of health inequality monitoring within their countries and regions. To this end, in some workshops, a subset of participants take part in additional training-of-trainers sessions, which prepare them to lead subsequent workshops and educational events within the country, thereby furthering knowledge dissemination.

#### Application and future plans

Workshop participants typically include: those working in national ministries of health, statistical offices, academia, and public health institutes; WHO colleagues working in headquarters, regional and countries offices; and participants from global health organizations like Gavi, the Vaccine Alliance and the Global Fund to Fight AIDS, Tuberculosis and Malaria. A prerequisite for enrolment is the ability to interpret, summarize and report data, and a working knowledge of health information.

Workshops have led to several key outputs. For example, workshops in Indonesia in 2016 were foundational to the development of the *State of health inequality: Indonesia* report [20]. This report was the product of a collaboration of stakeholders across several institutions, who were working to promote and build capacity for health inequality monitoring in the country [27]. The feedback and discussions arising during and after the workshops have also provided rich insights into participant needs, interests and abilities with regards to health inequality monitoring. These insights have been used to refine the workshops, and have informed subsequent activities (such as the development of the HEAT and HEAT Plus application [28, 29])﻿. There are plans to continue conducting workshops, including in response to requests from interested groups.

### Health Equity Assessment Toolkit (HEAT and HEAT Plus)

https://www.who.int/data/inequality-monitor/assessment_toolkit

#### Aim

HEAT and HEAT Plus is a free and open-source software application that facilitates the assessment of within-country health inequalities using disaggregated data [12]. The software was developed in response to a demand for a toolkit with the computational ability to display disaggregated data and summary measures of inequality in an interactive and customisable fashion. With functionality that supports the analysis, interpretation and reporting of health inequality data – including interactive data visualization – HEAT and HEAT Plus contributes to the broader aim of expanding and strengthening health inequality monitoring practices. The generation of evidence about health inequalities is a precursor for its integration into policy making processes.

#### Description

There are two editions of the toolkit. HEAT, Built-In Database Edition comes pre-installed with datasets from the WHO Health Inequality Data Repository (see below). HEAT Plus, Upload Database Edition allows users to upload and work with their own data. To upload data to HEAT Plus, the data must comply with a defined format (a template with a built-in validation tool is available to assist with the dataset preparation). Apart from the source of the data, both editions provide users with the same basic functionality: to explore patterns of inequality in disaggregated data; to calculate summary measures of inequality; to compare inequality between settings; and to create and export customized outputs. Examples of customized graph and map outputs are provided in an additional file (see Additional File 1).

The toolkit is organized around two main components. The ‘explore inequality’ component enables users to explore the situation in one setting of interest (for example, a country, province or district) to assess the latest situation of inequality and the change in inequalities over time. The ‘compare inequality’ component enables users to perform benchmarking, that is, compare the situation in one setting of interest with the situation in other settings. Within these components, the software provides options of analyzing inequalities using disaggregated data or summary measures of inequality, using a variety of graphs, maps and tables. Menu selections allow the data to be filtered according to setting, source, date, indicator and dimension of inequality. If the necessary information is available from the source data, the toolkit can display 95% confidence intervals, setting average, and population share of each subgroup.

#### Application and future plans

HEAT and HEAT Plus are primarily designed for use by those familiar with health information systems, and with basic skills in interpreting health-related data. This may include technical staff (for example, in ministries of health and statistical offices), public health professionals, researchers, and students. For instance, researchers may find value in the use of HEAT Plus, as they can upload their own datasets, calculate complex summary measures of inequality, and generate high-quality visual outputs of their analyses. The software serves as the default interface for the Health Inequality Data Repository and is used in conjunction with health inequality monitoring capacity building workshops. The toolkit is available in four languages (English, French, Portuguese and Spanish), with plans for further translation. Since its initial public launch in 2016, the software has undergone numerous updates and upgrades. Ongoing refinement of the toolkit in response to user feedback will continue.

#### Access

HEAT can be accessed and used online, while HEAT Plus is available as a downloadable desktop version for use offline. The software, along with user manuals, technical notes and the HEAT Plus template and validation tool, are available from the Health Inequality Monitor website [30].

### Data repository

https://www.who.int/data/inequality-monitor/data

#### Aim

The Health Inequality Data Repository, as of its April 2023 update, is the largest collection of publicly available disaggregated data about health and its determinants, including disaggregated data for all Sustainable Development Goal indicators where data are available (Kirkby K, Bergen N, Baptista A, Schlotheuber A, Hosseinpoor AR: Data resource profile: WHO Health Inequality Data Repository, under review). The data repository aims to support expanded health inequality monitoring by facilitating access to disaggregated datasets across multiple health topics and settings. The integration of (and compatibility of) datasets with the HEAT and HEAT Plus software application enables and promotes data exploration, analysis, benchmarking, and reporting.

#### Description

The updated data repository contains over 50 datasets, covering more than 2000 indicators and more than 25 dimensions of inequality across all world regions. In general, disaggregated data in the data repository tend to be reported at the country level or below, with an emphasis on indicators that are comparable across settings (that is, with comparable sources and calculation methods). Some of the topics featured in the Health Inequality Data Repository include, for example, reproductive, maternal, newborn and child health; COVID-19; HIV, tuberculosis and malaria; immunization; water, sanitation and hygiene; disability; burden of disease; social determinants of health; and nutrition. Accompanying metadata are available for all datasets. Regular updates to the data repository are planned to take place about once a year.

#### Application and future plans

The data repository is intended for use by diverse stakeholders with a range of technical skills and topical interests. By facilitating access to a large bank of disaggregated data, the data repository promotes the expanded use of data across different applications, including evidence-informed policymaking. All data contained within the data repository can be interactively explored online using HEAT, which provides a user-friendly interface for exploring patterns in disaggregated data, calculating summary measures of inequality and performing benchmarking between settings [30]. The datasets can also be downloaded for use with other analysis software programmes. This resource serves as an important data source for research. Data from the Health Inequality Data Repository are featured in a growing number of published works. The ongoing expansion of the scope of the datasets contained within the data repository will result in health inequality monitoring across new topic areas, dimensions of inequality and settings.

#### Access

All components of the data repository are freely accessible through the Health Inequality Monitor website [31].

### Other practical tools: Data source mapping templates and statistical codes

https://www.who.int/data/inequality-monitor/tools-resources

#### Aim

Other practical tools have been developed, including data source mapping templates and statistical codes, with the aim of streamlining the steps of health inequality monitoring. The data source mapping templates and statistical codes are applicable to steps 2 and 3, respectively, of the health inequality monitoring cycle (Fig. 1).

#### Description

Data source mapping templates are worksheets for mapping data availability for health inequality monitoring. The worksheets allow users to organize information about available data sources and assess possibilities for use in inequality monitoring, including linking data between two or more sources. The templates can be downloaded and modified for use. Statistical codes are provided for selected statistical packages that are commonly used for health inequality monitoring: R, SAS, SPSS and Stata. They demonstrate how complex survey sampling design can be taken into account in the calculation of disaggregated health data and population subgroup sizes, the two pieces of information that are important for calculating summary measures of inequality.

#### Application and future plans

These resources are practical tools, primarily intended for those who are actively engaged in carrying out health inequality monitoring. As such, they are of particular interest to analysts, researchers and students. These tools will continue to be updated and expanded in response to challenges or inefficiencies that arise in the application of health inequality monitoring.

## Conclusions

WHO has made several important contributions to the area of health inequality monitoring, including: a global repository of disaggregated data; an interactive toolkit for assessing, analysing and reporting health inequalities; global state of inequality reports; training opportunities; and practical tools and resources. These outputs encompass a variety of functions, formats and delivery modalities with the goal of making them accessible and applicable for diverse target audiences to fulfill different purposes. WHO activities, resources and tools for health inequality monitoring are now embedded within the Inequality Monitoring and Analysis Strategy, which articulates goals and targets as well as an implementation roadmap and monitoring and evaluation framework. This reinforces a key strength of these contributions – that they reflect a common underlying approach to inequality monitoring that is systematic and can be developed, adapted and applied across topics, settings and populations. Thus, these different resources and tools are mutually reinforcing, with wide relevance within both health and non-health sectors. This work is strengthened through close collaborations with partners within and outside of WHO.

A dedication to improving inequities in health and its determinants is a longstanding priority of WHO and global health and development partners. With prominent global targets and goals emphasizing the importance of advancing equity in health, there is a need for high-quality evidence on health inequalities across diverse topics and populations to support evidence-informed, equity-oriented policy making. Accordingly, monitoring health inequalities is of growing importance within a wide array of health topics as well as topics related to the determinants of health and wellbeing, including those captured by Sustainable Development Goal monitoring frameworks.

## Supplementary Information

**Additional file 1.** Examples of customized graph and map outputs generated by the WHO Health Equity Assessment Toolkit: (a) horizontal line graph; (b) vertical bar graph; (c) horizontal bar graph; (d) map.

## Data Availability

Where applicable, the tools and resources outlined in this manuscript are publicly available from the Health Inequality Monitor website (https://www.who.int/data/inequality-monitor), and/or other channels mentioned in the article text.

## Abbreviations

GPW 13: World Health Organization Thirteenth General Programme of Work
HEAT: Health Equity Assessment Toolkit
WHO: World Health Organization

## References

[1] Chisholm B (1949). Social medicine. Sci Am.

[2] United Nations General Assembly (2015). Transforming our world: the 2030 Agenda for Sustainable Development.

[3] World Health Organization. Triple Billion dashboard [Internet]. 2022 [cited 2022 Nov 10]. Available from: https://www.who.int/data/triple-billion-dashboard.

[4] World Health Organization (2019). The Thirteenth General Programme of Work, 2019–2023.

[5] World Health Organization (2021). SCORE for health data technical package: global report on health data systems and capacity, 2020.

[6] World Health Organization (2013). Handbook on health inequality monitoring: with a special focus on low-and middle-income countries.

[7] World Health Organization (2017). National health inequality monitoring: a step-by-step manual.

[8] World Health Organization. Health equity [Internet]. 2022 [cited 2022 Nov 10]. Available from: https://www.who.int/health-topics/health-equity#tab=tab_1.

[9] World Health Organization. Health equity and its determinants. World Health Day (2021). it’s time to build a fairer, healthier world for everyone, everywhere.

[10] Hosseinpoor AR, Bergen N, Schlotheuber A. Promoting health equity: WHO health inequality monitoring at global and national levels. Glob Health Action. 2015;8(1654–9880 (Electronic)):29034. 10.3402/gha.v8.29034 PMC4576419 26387506

[11] World Health Organization. Inequality monitoring and analysis strategy 2022–27 [Internet]. Geneva: World Health Organization; 2022 [cited 2022 Nov 10]. Available from: https://www.who.int/publications/m/item/inequality-monitoring-and-analysis-strategy-2022-27.

[12] Kirkby K, Schlotheuber A, Vidal Fuertes C, Ross Z, Hosseinpoor AR (2022). Health Equity Assessment Toolkit (HEAT and HEAT Plus): exploring inequalities in the COVID-19 pandemic era. International Journal for Equity in Health.

[13] World Health Organization (2019). Inequality monitoring in immunization: a step-by-step manual.

[14] World Health Organization (2022). Inequality monitoring in sexual, reproductive, maternal, newborn, child and adolescent health: a step-by-step manual.

[15] World Health Organization. Health inequality monitoring workbook: exercises to guide the process of health inequality monitoring [Internet]. Geneva: World Health Organization; 2022 [cited 2022 Nov 10]. Available from: https://apps.who.int/iris/bitstream/handle/10665/358893/WHO-DDI-DNA-MFI-2022.3-eng.pdf.

[16] World Health Organization. Companion workbook: exercises to guide the process of inequality monitoring in sexual, reproductive, maternal, newborn, child and adolescent health [Internet]. Geneva: World Health Organization; 2022 [cited 2022 Nov 10]. Available from: https://www.who.int/publications/i/item/WHO-DNA-MCA-SRH-2022.1.

[17] World Health Organization. Tools and resources for health inequality monitoring [Internet]. Health Inequality Monitor. 2022 [cited 2022 Nov 16]. Available from: https://www.who.int/data/inequality-monitor/tools-resources.

[18] World Health Organization (2015). State of inequality: reproductive, maternal, newborn and child health.

[19] World Health Organization (2016). State of inequality: childhood immunization.

[20] World Health Organization (2017). State of health inequality: Indonesia.

[21] World Health Organization (2021). State of inequality: HIV, tuberculosis and malaria.

[22] World Health Organization (2018). Explorations of inequality: childhood immunization.

[23] World Health Organization. Publications [Internet]. Health Inequality Monitor. 2022 [cited 2022 Nov 16]. Available from: https://www.who.int/data/inequality-monitor/publications.

[24] Bergen N, Kirkby K, Baptista A, Nambiar D, Schlotheuber A, Vidal Fuertes C (2022). Health Inequality Monitoring channel on OpenWHO: capacity strengthening through eLearning. Int J Equity Health.

[25] World Health Organization. Training [Internet]. Health Inequality Monitor. 2022 [cited 2022 Nov 16]. Available from: https://www.who.int/data/inequality-monitor/training.

[26] World Health Organization. Health inequality monitoring [Internet]. OpenWHO. 2022 [cited 2022 Nov 16]. Available from: https://openwho.org/channels/inequality-monitoring.

[27] Hosseinpoor AR, Nambiar D, Tawilah J, Schlotheuber A, Briot B, Bateman M (2018). Capacity building for health-inequality monitoring in Indonesia: enhancing the equity orientation of country health information system. Glob Health Action.

[28] Hosseinpoor AR, Nambiar D, Schlotheuber A, Reidpath D, Ross Z (2016). Health Equity Assessment Toolkit (HEAT): software for exploring and comparing health inequalities in countries. BMC Med Res Methodol.

[29] Hosseinpoor AR, Schlotheuber A, Nambiar D, Ross Z (2018). Health Equity Assessment Toolkit Plus (HEAT Plus): software for exploring and comparing health inequalities using uploaded datasets. Glob Health Action.

[30] World Health Organization. Health Equity Assessment Toolkit [Internet]. Health Inequality Monitor. 2022 [cited 2022 Nov 16]. Available from: https://www.who.int/data/inequality-monitor/assessment_toolkit.

[31] World Health Organization. Data Repository [Internet]. Health Inequality Monitor. 2022 [cited 2022 Nov 16]. Available from: https://www.who.int/data/inequality-monitor/data.

