# Supplementary material for: Strengthening and expanding health inequality monitoring for the advancement of health equity: a review of WHO resources and contributions
Source: Int J Equity Health. 2023 Mar 17;22:49. doi: 10.1186/s12939-022-01811-4 (PMC10022555; doi:10.1186/s12939-022-01811-4)

**Additional File 1.** Examples of customized graph and map outputs generated by the WHO Health Equity Assessment Toolkit: (a) horizontal line graph; (b) vertical bar graph; (c) horizontal bar graph; (d) map

1. Horizontal line graph


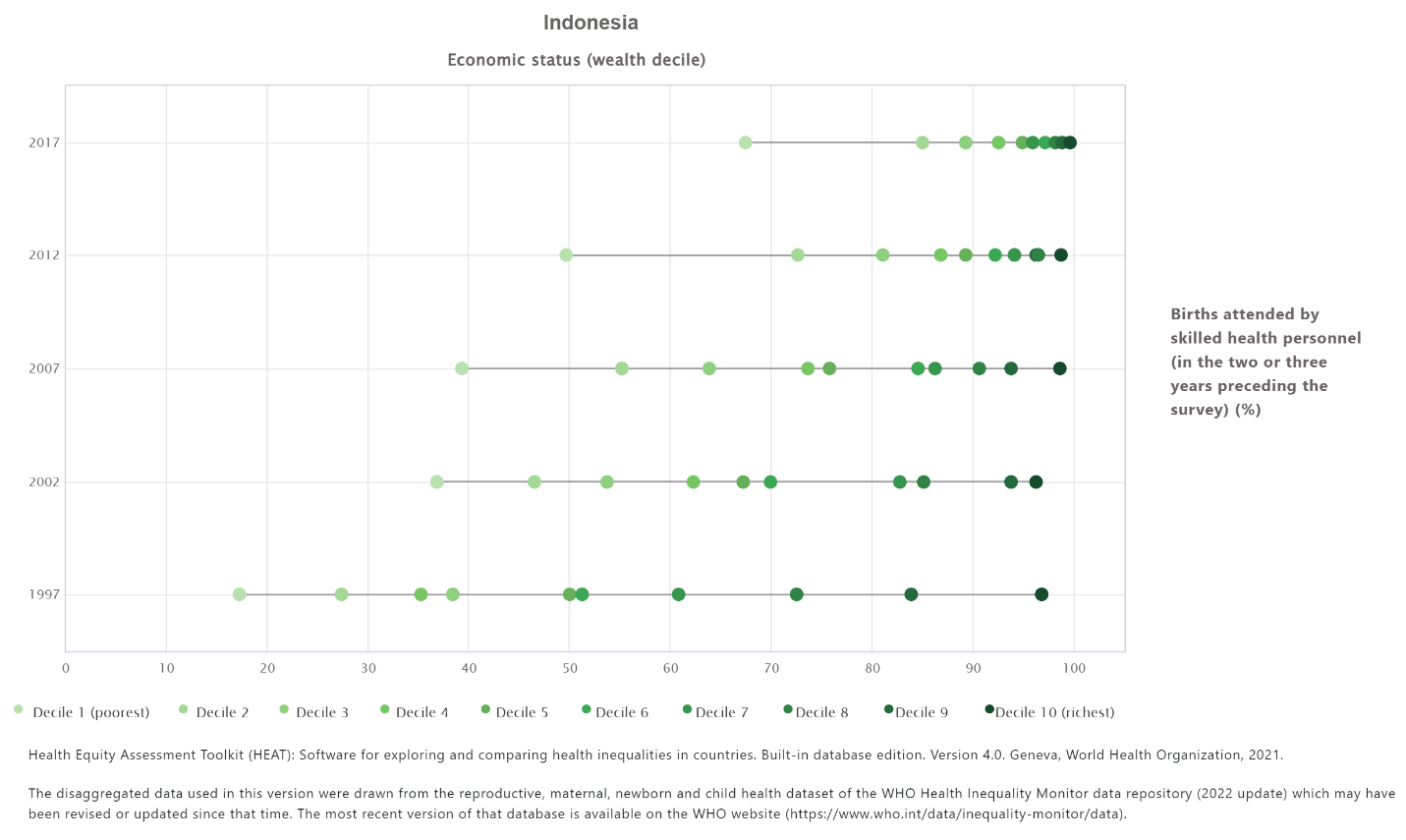


1. Vertical bar graph


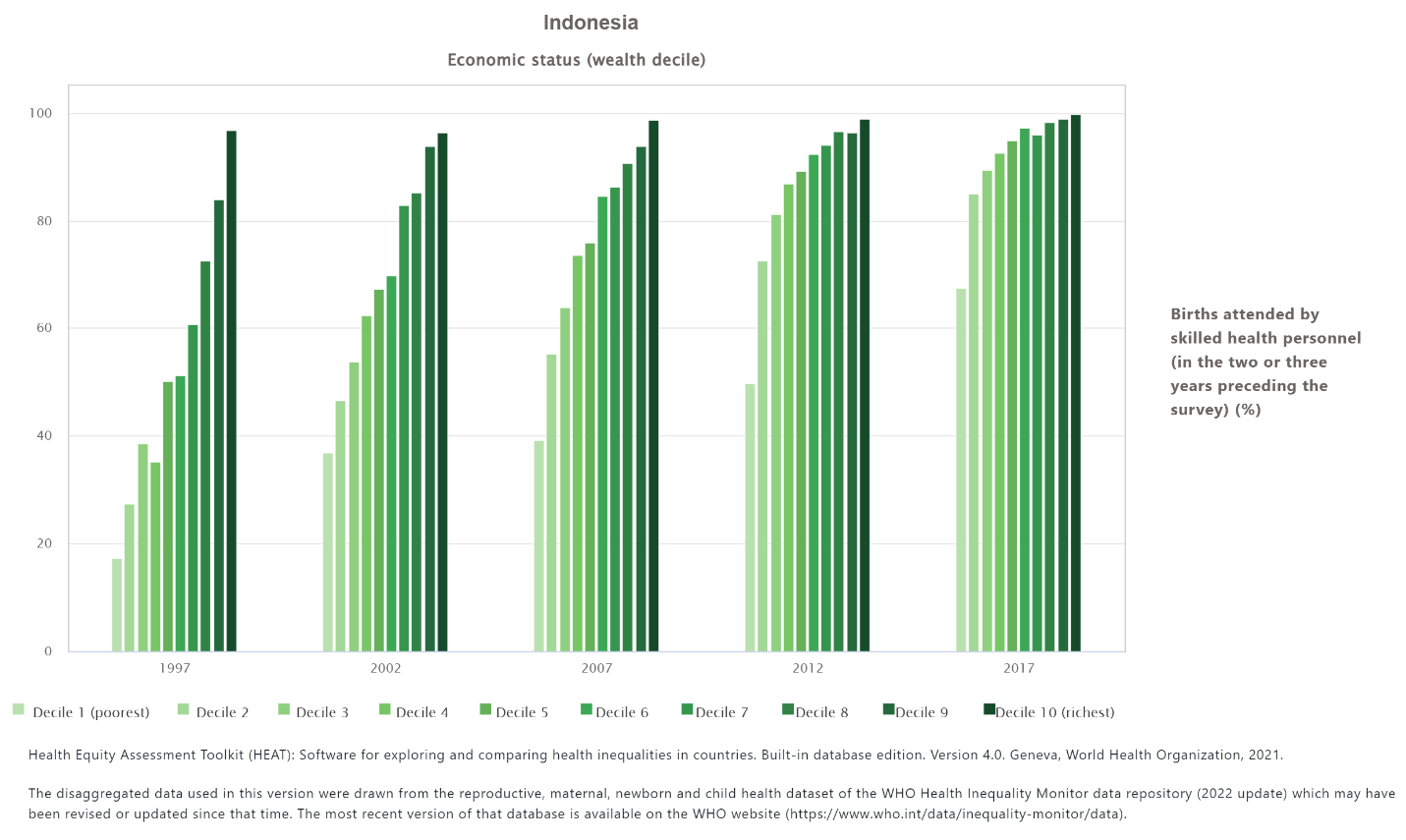


1. Horizontal bar graph


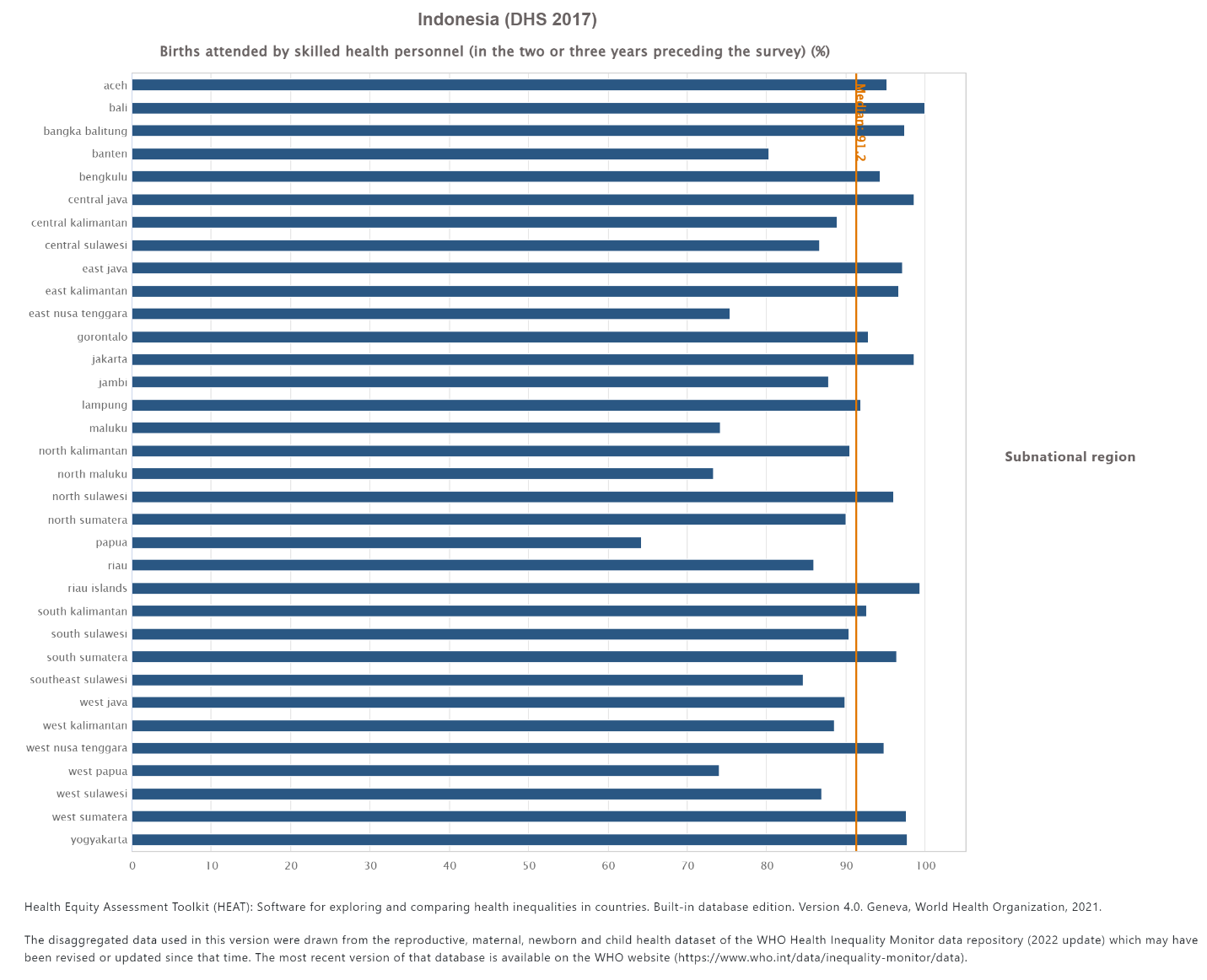


1. Map


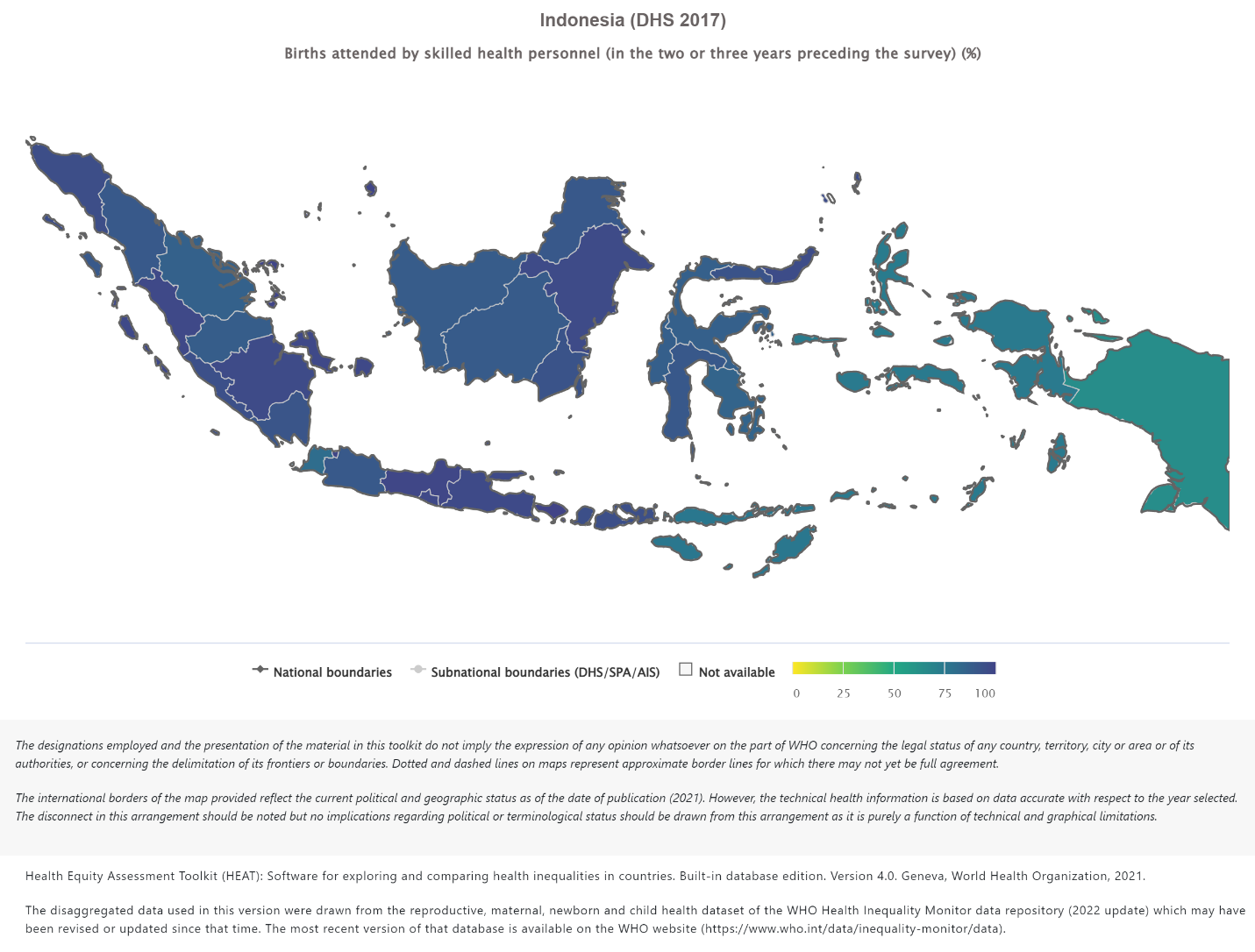

Supplement: Supplementary file 1 — Additional file 1. Examples of customized graph and map outputs generated by the WHO Health Equity Assessment Toolkit: (a) horizontal line graph; (b) vertical bar graph; (c) horizontal bar graph; (d) map. [file 12939_2022_1811_MOESM1_ESM.docx]
